# In Premature Newborns Intraventricular Hemorrhage Causes Cerebral Vasospasm and Associated Neurodisability *via* Heme-Induced Inflammasome-Mediated Interleukin-1 Production and Nitric Oxide Depletion

**DOI:** 10.3389/fneur.2017.00423

**Published:** 2017-08-18

**Authors:** Michael Eisenhut, Samyami Choudhury

**Affiliations:** ^1^Pediatric Department, Luton and Dunstable University Hospital NHS Foundation Trust, Luton, Bedfordshire, United Kingdom

**Keywords:** hemorrhage, vasospasm, heme, inflammasome, nitric oxide, interleukin-1

## Abstract

**Background:**

Intraventricular hemorrhage (IVH) occurs in 60–70% of neonates weighing 500–750 g and 10–20% of those weighing 1,000–1,500 g. All forms of IVH have been associated with neurocognitive deficits. Both subarachnoid and IVHs have been associated with delayed vasospasm leading to neurological deficits. Pathways linking hemoglobin release from blood clots to vasospasm include heme-induced activation of inflammasomes releasing interleukin-1 (IL-1) that can cause calcium dependent and independent vasospasm. Free hemoglobin is a potent scavenger of nitric oxide (NO). Depletion of NO, a potent endogenous vasodilator, has been associated with features of vasospasm.

**Hypothesis:**

In premature newborns, IVH causes cerebral vasospasm and associated neurodisability *via* heme-induced increased inflammasome-mediated IL-1 production and NO depletion.

**Confirmation of hypothesis and implications:**

This hypothesis could be confirmed in the IVH animal model with visualization of any associated vasospasm by angiography and in newborns with IVH by transcranial Doppler ultrasonography and correlation with cerebrospinal fluid IL-1 and NO metabolite levels. Confirmation of the role of heme in activation of inflammasomes causing IL-1 production and NO binding could be achieved by measuring the effect of heme scavenging interventions on IL-1 levels and levels of NO metabolites. In addition to removal of the accumulated blood of an IVH by drainage, irrigation, and fibrinolytic therapy intrathecal application of vasodilators and heme scavenging agents like haptoglobin and haemopexin and systemic treatment with inhibitors of inflammasomes like telmisartan could be used to prevent and treat cerebral vasospasm, and thus reduce the risk of associated brain injury in premature neonates.

## Introduction

Arterial development is completed initially in brainstem and cerebellum (20–24 weeks) followed by the basal ganglia and diencephalon by 24–28 weeks and finally the cortex and germinal matrix. Rupture of fragile capillaries in the subependymal germinal matrix can give rise to intraventricular hemorrhage (IVH). Immaturity of the cerebral vasculature in the border zones between vascular territories makes the preterm brain vulnerable to bleeding (1). The immature vasoregulation coupled with rises in arterial pressure due to the stresses of postnatal adaption after extreme premature delivery contributes to the pathogenesis of IVH. IVH occurs 12–72 h after birth (2). IVH causes significant morbidity and mortality in the preterm infant group. IVH occurs in 60–70% of neonates weighing 500–750 g and 10–20% of those weighing 1,000–1,500 g (3). IVH can be divided into four degrees of severity according to Papile (4):

- Grade I—bleeding occurs just in the germinal matrix.
- Grade II—bleeding also occurs inside the ventricles, but they are not enlarged.
- Grade III—ventricles are enlarged by the accumulated blood.
- Grade IV—bleeding extends into the brain tissue around the ventricles.

Prematurity and low birth weight are the most important risk factors for grade IV hemorrhage. Other risk factors for IVH identified include the absence of prenatal steroid therapy in woman at risk of premature delivery, symptoms of intrauterine infection in the newborn, treatment with fluid boluses and catecholamines for hypotension and treatment for metabolic acidosis, blood clotting disorders, thrombocytopenia, and hypoglycemia. Most of the mentioned risk factors are associated with a systemic inflammatory response involving cytokine release (5). Preterm infants with grade IV IVH are at the high risk of developing neuropathological complications including posthemorrhagic hydrocephalus, periventricular hemorrhagic infarction, germinal matrix destruction, and periventricular leukomalacia. This leads to significant neurodevelopmental morbidity. In one case–control study of 1,472 infants with a history of preterm delivery (23–28 weeks gestational age at birth), those with grade III–IV IVH had significantly increased rates of cerebral palsy (30%), developmental delay (17.5%), deafness (8.6%), and blindness (2.2%). Infants with grade I–II IVH also had compared with infants without IVH increased rates of neurosensory impairment (22 vs 12.1%), cerebral palsy (10.4 vs 6.5%), developmental delay (7.8 vs 3.4%), and deafness (6.0 vs 2.3%) (1). The fact that lower degrees of IVH are also associated with significant neurological morbidity was confirmed by Patra et al. who found that infants who have extremely low birth weight with grades I–II IVH had poor neurodevelopmental outcomes at 20 months than infants with normal cranial ultrasound (6).

The consequences of extravasated blood around blood vessels are evident from what we have learnt about the pathophysiology of brain injury in subarachnoid hemorrhage (SAH) (7, 8). Extravasated red blood cells release on disintegration free hemoglobin which acts as a nitric oxide (NO) scavenger (7). NO, which is generated from l-arginine by endothelial NO synthase stimulates soluble guanylate cyclase in vascular muscle resulting in an increase in the intracellular concentration of guanosine 3′,5′-cyclic monophosphate resulting in relaxation of vascular smooth muscle (9). NO depletion subsequently leads to vasoconstriction causing ischemia and subsequent tissue injury (7).

Free heme and tissue injury trigger a localized inflammatory response involving inflammasome namely, nucleotide-binding and oligomerization domain-like receptor pyrin domain-containing protein 3 (NLRP3) activation (10). Heme activates NLRP3 through P2X receptors, especially the P2X7R and P2X4R. The molecular mechanism by which heme promotes NLRP3 activation in the presence of serum requires Syk phosphorylation, reactive oxygen species, and K^+^ efflux. NLRP3 activation then leads to increased production of interleukin-1 (IL-1) in macrophages. All forms of neuroinflammation that involves production of IL-1 have been, where this has been investigated, shown to be associated with cerebral vasospasm (11).

Interleukin-1 induces the production of IL-6. Increased concentrations of IL-6 in the cerebrospinal fluid were found to be associated with white matter injury in premature infants (12).

Interleukin-1 has been shown to cause vasoconstriction by calcium dependent and independent mechanisms. Investigations in patients with SAH showed that CSF levels of IL-1-beta were increased and correlated with the later development of cerebral vasospasm. IL-1 acts through G-protein-coupled receptors by activation of intracellular phospholipase C and phospholipase mediated protein kinase C (PKC) activation (11):

1. Phosphatidylinositol trisphosphate produced by phospholipase C activity leads to calcium release that causes myosin light chain (MLC) kinase activation triggering MLC activation and calcium-dependent vasospasm.
2. Activation of PKC through diacylglycerol generated by phospholipase C action from phosphatidylinositol (4,5) bisphosphate can act through phosphorylation and hence activation of the MLC kinase. Prolonged contraction of blood vessels lasting up to 2 weeks has been found to be related to this mechanism.
3. MLC phosphorylation subsequent to PKC activation is increased through activation of rho-kinase and the myosin-binding subunit (MBS) of MLC phosphatase (MLCPh). Rho-kinase hereby phosphorylates MBS, which results in the inhibition of MLCPh. The reduced MLCPh activity is associated with increased phosphorylation and hence contractility of the MLC resulting in calcium-independent vasospasm. There is a highly significant correlation between the extent of MBS phosphorylation and contractions.

## Hypothesis

In premature newborns, IVH causes cerebral vasospasm and associated neurodisability *via* heme-induced increased inflammasome-mediated IL-1 production and NO depletion.

### Explanation of Hypothesis

This hypothesis states that the effects of breakdown products of extravasated red blood cells cause contraction of blood vessels at the arterial side of the circulation and that this contraction causes a lack of blood supply leading to neuronal cell death causing permanent brain injury and resulting neurodisability. Previous theories attributed brain injury after hemorrhage mainly to ischemia from rupture of nutrient vessels, stasis of blood flow from disruption of venous drainage, ischemia from a pressure effect exerted by the extravasated blood or blockage of cerebrospinal fluid drainage, and cytotoxic effects of the posthemorrhagic inflammatory response (13, 14).

These effects may be present and contributory to brain damage but are with the exception of measures to reduce intracranial pressure largely inaccessible to therapeutic interventions and reversible factors like vasospasm need to be explored to find ways of improving outcome. Vasospasm is a reversible cause of ischemia and can be addressed by preventative and therapeutic interventions.

## Evidence to Support the Hypothesis

- In patients with SAH, additional grade IV IVH is significantly associated with ultra-early angiographic vasospasm, which was associated with symptoms, delayed cerebral ischemia and infarction, unfavorable outcome at follow-up, and greater mortality (15).
- Using transcranial Doppler ultrasound evidence of cerebral vasospasm has been detected in all forms of IVH, including those arising from hypertension under the influence of anticoagulant and antithrombotic agents and arteriovenous malformations (AVM). This supports the hypothetical assumption that not the process causing the bleeding but the blood itself is involved in causing the cerebral vasospasm. In adults above 50 years of age, cerebral vasospasm was common in IVH and occurred in 62.9% of patients (*n* = 62) on serial transcranial Doppler ultrasound scanning that was performed at 48 h between day 4 and 6 and between day 7 and 9 after diagnosis of IVH including insonation of the middle, anterior, and posterior cerebral arteries as well as the siphon, vertebral arteries in the V4 segment and the basilar artery with a grading according to peak systolic velocity (16). In a study using stricter criteria for cerebral vasospasm including in addition to the velocity criteria, the Lindegaard ratio (ratio > 3 in middle cerebral artery flow to internal carotid artery flow as indicator of cerebral vasospasm) about 6% of patients with IVH were found to have vasospasm (17).
- Detailed descriptions of the characteristics of vasospasm and treatment attempts are available from case reports. There are 13 previous reports of cerebral vasospasm associated with IVH as complication of ruptured AVMs including several without SAH (see Table 1). In one child with IVH early fever was found to be a predictor of vasospasm similar to what has been observed in patients with SAH, which supports the role of inflammation and the endogenous pyrogen IL-1 in causing the vasospasm found (18). The lack of success of calcium antagonists in reducing vasospasm as reported in case reports (see Table 1) and lack of influence on outcome of intracerebral bleeds may be due to the fact that IL-1 caused vasospasm predominantly through a calcium-independent pathway.
- If inflammasome-induced IL-1 causes vasospasm-induced brain injury, there should be brain injury associated with sterile brain inflammation in preterm neonates with a systemic inflammatory response syndrome not associated with meningitis. This has been indeed confirmed. Preterm neonates with early onset clinical sepsis without meningitis had in 22% evidence of white matter damage on MRI. The neonates with death or abnormal neuroimaging as outcome had significantly higher cerebrospinal IL-1 levels (19).
- The effect of IVH on vasospasm in arteries remote from the location of the IVH is consistent with one or more mediators carried by the CSF and transmitting this effect from the blood clot to the arteries. The delayed onset of vasospasm after IVH would be consistent with a threshold of the concentration of mediators in the CSF above or below which vasoconstriction occurs. Vasospasm could also depend on both duration of effect and quantity of the mediator involved. The CSF circulation could hereby modulate the concentration of any mediators involved; Black previously reported a significant association between hydrocephalus and vasospasm in patients with SAH; in 62% of cases, vasospasm and hydrocephalus occurred concomitantly and in 11% of patients, vasospasm was not associated with hydrocephalus (20). The fact that the quantity of the mediator is crucial in triggering the vasospasm is supported by the observed correlation of the quantity of blood injected in an experimental model of SAH and vasospasm (21).
- The potent vasodilator inhaled NO given to support pulmonary blood flow and improve oxygenation in newborns with severe respiratory failure has been shown to improve neurodevelopmental outcome at 2 and at over 5 years of age mainly due to a 47% decrease in the risk of cognitive impairment (defined by a score of less than 70 on the Bayley Mental Developmental Index) (*p* = 0.03) (22, 23).
- The potent smooth muscle relaxant intravenous magnesium sulfate when given antenatally to mothers is partially responsible for reduction of ultrasonic evidence of cerebral injury in preterm infants less than 32 weeks gestation and cerebral palsy at 2 years of age (24).

**Table 1:** Characteristics of patients with intraventricular hemorrhage (IVH) as complication of arteriovenous malformation who had cerebral vasospasm detected.

Age (years)	Type of hemorrhage	Method involved in detection of vasospasm	Blood vessel involved in vasospasm	Timing of vasospasm	Medication used to treat vasospasm and effect	Features of neurodisability	Reference
11	Isolated IVH in lateral, third, and fourth ventricle	Cerebral angiography	A1 and M1 segments of both anterior and middle cerebral arteries	Day 4	Corticosteroids, osmotherapy with glycerol without effect	Right sided hemiparesis, speech disturbance and right hemianopsia	Yanaka et al. (25)

31	IVH and intracerebral hemorrhage in corpus callosum	Cerebral angiography	Supraclinoid portion of bilateral internal carotid arteries, bilateral anterior, and middle cerebral arteries	Day 13	Papaverine, intravenous nimodipine without effect	Hydrocephalus, bilateral motor weakness	Park et al. (26)

41	Isolated IVH in both lateral ventricles	Magnetic resonance angiography, cerebral angiography	Bilateral middle cerebral arteries, right anterior cerebral artery, left supraclinoid internal carotid artery, basilar artery, and bilateral posterior cerebral arteries	Day 10	Verapamil intra-arterial in right and left internal carotid with immediate clinical improvement and vertebral arteries, nimodipine	Right hemineglect, left-gaze preference, mild right-facial droop	Gerard et al. (27)

33	Isolated IVH in lateral and fourth ventricles	MR angiography	Supraclinoid portions of the bilateral internal carotid arteries and the supraclinoid, A1 and distal portions of the anterior cerebral arteries	Day 17	Intravenous calcium antagonists, radical scavengers, and volume expanders with no success	Reduced verbal response and motor aphasia, mild right sided hemiparesis	Yokobori et al. (28)

37	IVH in all four ventricles and thalamic bleed	Perfusion CT	Diffuse cerebral vasospasm	Day 10	Intravenous then oral nimedipine, intravenous norepinephrine, intrathecal urokinase, and ventricular drainage without effect	Transient left sided hemiparesis and speech impediment	Tseng and Tsai (29)

40	Right thalamic and IVH in all four ventricles	Cerebral angiogram	Supraclinoid portion of both internal carotid arteries	Day 6	Induced hypertension with volume expansion	Persistent right sided mild hemiparesis	Maeda et al. (30)

11	Right putaminal hemorrhage and bleed in all four ventricles	Angiogram	Supraclinoid portion of both internal carotic arteries	Day 17	No data	Persistent mild left sided hemiparesis	Maeda et al. (30)

44	IVH of the fourth ventricle, right lateral ventricle	Cerebral artery angiography	Left posteroinferior cerebellar artery left and right vertebral arteries	Day 3	Hypervolemia with intravenous fluids, induction of hypertension with neosynephrine with a mean arterial blood pressure goal of 130–140 mmHg for 4 days with clear resolution of neurological symptoms in response to it	Left facial paralysis, left beating nystagmus, vertigo, slurred speech	Dull and Torbey (31)

26	IVH in the right lateral ventricle	Cerebral angiography	Middle and anterior cerebral arteries	Day 17	No data	Left homonymous hemianopsia	Kobayashi et al. (32)

31	IVH and hemorrhage in the head of the right caudate nucleus and the genu of the corpus callosum	Cerebral angiography, transcranial Doppler ultrasound	Both internal carotid arteries	Day 16	Transluminal angioplasty of the right internal carotid artery with a single-lumen, over-the-wire balloon catheter with clear effect on vasospasm, intravenous nimodipine	Left sided hemiparesis, neuropsychological deficits	Kothbauer et al. (33)

5	Isolated IVH	Cerebral angiography, transcranial Doppler ultrasound	Bilateral internal, middle, and anterior cerebral arteries, left proximal posterior cerebral artery	Day 10	Hypervolemia and hypertension aiming at blood pressure of 180 and nimodipine	Fixed and dilated left pupil, deviation of the eyes to the left and reduced level of consciousness to GCS 9	Spader et al. (18)

13	Isolated predominantly left sided and fourth ventricle IVH	MR angiography, conventional cerebral angiography	Anterior and medium cerebral artery	Day 6	Intra-arterial nicardipine injection, baloon angioplasty daily	Right sided hemiparesis	Pendharkar et al. (34)

59	Combined bilateral IVH in lateral ventricles and subarachnoid hemorrhages	Transcranial Doppler ultrasound	General vasospasm	Day 2	Nimodipine treatment	None	De Carvalho (35)

## Evidence Against the Hypothesis

- A recent Cochrane review on the impact of the vasodilator inhaled NO on the outcome of respiratory failure in preterm neonates found no impact of its use on long-term neurodisability (36).
- Any effects of NO on neurodisability may not have been due to influence on vasospasm but simply have been due to improved cerebral oxygenation (37).
- The influence of magnesium sulfate on cerebral lesions and cerebral palsy may have been due to improved placental perfusion (38).
- Intravenous application of the vasodilating calcium antagonist nicardipine lowering blood pressure in patients with intracerebral hemorrhage and able to penetrate the blood–brain barrier did not improve neurological outcome in adults (39).

## Testing of the Hypothesis

Before embarking on trials of intervention cerebral vasospasm in neonatal IVH needs to be documented and assessed in longitudinal studies at the timepoints at which post-IVH cerebral vasospasm has been detected in adults namely at 48 h, between day 4 and 6 and between day 7 and 9 after diagnosis of IVH. To assess the presence of cerebral vasospasm in preterm neonates, non-invasive methods need to be employed. Blood velocity measurements using transcranial Doppler ultrasound can be used in preterm neonates for detection of cerebral vasospasm. The Lindegaard ratio (described earlier) can again be used to diagnose vasospasm (40). The long-term neurological outcome needs to be compared between neonates with and without cerebral vasospasm detected by this method.

Future studies could measure NO metabolites and IL-1 in the CSF of preterms with IVH serially (e.g., daily) to determine whether the increase (IL-1) or decrease (NO metabolites) in concentration over time could predict the crossing of a “vasospasmogenic threshold” and thus predict cerebral vasospasm. The absolute concentration could in this context also be correlated with the time to evolution of the vasospasm. Heme or its metabolite bilirubin could be measured at the same time to assess its predictive value for vasospasm and the need for preventative intervention.

## Implications of a Confirmation of the Hypothesis

The most important intervention with significant future potential in reduction and prevention of cerebral vasospasm and its associated neurodisability in neonatal IVH is rapid removal of the accumulated blood by all available means. Delayed removal of a perivascular clot in SAH has been demonstrated to be associated with a lack of resolution of vasospasm since clot removal up to 3 days after hemorrhage reduced vasospasm, but after 5 days, it had little influence (41). Drainage, irrigation, and fibrinolytic therapy (DRIFT) in infants with posthemorrhagic ventricular dilatation followed up until 2 years of age showed the following results: of 39 infants assigned to DRIFT, 21 (54%) died or were severely disabled vs 27 of 38 (71%) in the standard group (adjusted odds ratio 0.25 [95% confidence interval: 0.08–0.82]). Among the survivors, 11 of 35 (31%) in the DRIFT group had severe cognitive disability vs 19 of 32 (59%) in the standard group (adjusted odds ratio: 0.17 [95% confidence interval: 0.05–0.57]). Median Mental Development Index was 68 with DRIFT and <50 with standard care. Severe sensorimotor disability was not significantly reduced (42). Some of this effect may have been due to treatment of the evolving hydrocephalus but removal of the ongoing heme sources scavenging NO and causing ongoing release of vasospasm causing IL-1 may have been crucial. To assess the impact of the DRIFT intervention in all grades of IVH, ultrasound Doppler studies assessing correlates of vasospasm before and after the intervention need to be explored and put into relationship to neurological long-term outcome. At the same time, one could measure peripheral blood glial fibrillary acidic protein levels—a sensitive marker of brain injury—before and after the intervention and in controls without the intervention. Neuroendoscopic lavage for clot removal has been successfully and safely employed and shown to be possibly superior to DRIFT related procedures in preventing hydrocephalus and warrants further study (43).

Optimization of cerebral perfusion pressure by hypervolemia with intravenous fluids and induction of hypertension with neosynephrine appears to be an important auxiliary measure to be put into place as anecdotal evidence suggests (see Table 1).

As the listed case reports demonstrate larger cerebral arteries can be involved in IVH-associated vasospasm. Angioplasty has been successful in resolution of vasospasm in SAH and IVH (44, 45) and needs to be explored in established vasospasm in preterm neonates.

Case reports support the future use of peripheral intravenous infusion of the NO donor molsidomine, and intrathecal application of nitroprusside sodium in patients with IVH because they appear to reduce cerebral vasospasm (46).

To support DRIFT once established as an effective intervention intrathecal adjuvant high-dose vasodilator, anti-inflammatory, and hemoglobin and heme scavenging treatments should be employed using slow release preparations where available. Such drugs could include tissue plasminogen activator or NO donors in isolation or in combination (47) and heme scavengers haemopexin and haptoglobin (48, 49). One may argue that conclusions from the data on vasospasm in IVH in adults are not transferable to preterm neonates because inflammation may be different in the fetus and therefore the preterm infant due to the immaturity of the immune system. It is, however, unclear whether this immaturity affects IL-1 production following IVH: experiments employing lipopolysaccharide-induced IL-1 secretion from very low birth weight infants (VLBWI) and from newborns after 30 weeks gestation in comparison to adults showed a diminished *ex vivo* LPS stimulated IL-1 beta secretion into whole blood in VLBWI (50). In line with these findings Brochu et al. found a weaker IL-1beta response in the immature compared to the more mature term-like brain. In this rat model, as well as in human preterm newborns, the IL-1b response takes place mainly in deep white matter that corresponds to the area of vulnerability to IVH. Prematurity may be associated with an imbalance of the IL-1beta/IL-1receptor antagonist (IL-1-Ra) ratio due to a reduction of upregulation of IL-1Ra expression (51, 52). This may mean a reduced expression of IL-1 in response to stimuli is at least partially offset by an even more reduced anti-IL-1 response. Whole genome analysis of the transcriptome of the fetal inflammatory response syndrome in umbilical cord blood from preterm neonates with fetal inflammatory response syndrome revealed on microarray analysis of leukocyte RNA, a substantial degree of similarity with the systemic inflammatory response syndrome in adults (53).

To inhibit the neuroinflammation associated with heme-induced activation of inflammasomes and with increased IL-1 production leading to an increased risk of vasospasm one could treat the affected newborns with inflammasome inhibitors like telmisartan which has been shown to reduce NLRP3 activity and IL-1 levels and associated cerebral injury (54–56). These anti-inflammatory interventions could be safe in all degrees of severity of IVH but need to be first explored in animal studies. The intervention to reduce vasospasm-associated neurodisability should be employed as soon as an IVH is detected to act as effective preventative. Such animal experiments also need to explore the effect of intra-arterial verapamil, which has been shown to relieve vasospasm in a patient with IVH (see Table 1). All drug treatment interventions including intrathecal application could be assessed in the preterm rabbit model of glycerol-induced IVH, which has been developed for this purpose (57). Such studies could analyze the impact of inflammasome inhibitors and NO donors on transcranial Doppler-ultrasonographic evidence of vasospasm on neurological symptoms and histological pattern of brain injury.

## Author Contributions

ME conceived the hypothesis, drafted the outline of the review, and wrote the final version of the manuscript. SC made substantial contributions to the acquisition of data and revision. Both authors gave the final approval of the version to be published, agreed to be accountable for all aspects of the work in ensuring that analysis or interpretation of data for the work, and agreed to be accountable for all aspects of the work in ensuring that questions related to the accuracy or integrity of any part of the work are appropriately investigated and resolved.

## Conflict of Interest Statement

The authors declare that the research was conducted in the absence of any commercial or financial relationships that could be construed as a potential conflict of interest.
